# CircESRP1 enhances metastasis and epithelial–mesenchymal transition in endometrial cancer via the miR-874-3p/CPEB4 axis

**DOI:** 10.1186/s12967-022-03334-6

**Published:** 2022-03-22

**Authors:** Rui Shi, Wei Zhang, Jun Zhang, Zhicheng Yu, Lanfen An, Rong Zhao, Xing Zhou, Ziwei Wang, Sitian Wei, Hongbo Wang

**Affiliations:** Department of Obstetrics and Gynecology, Union Hospital, Tongji Medical College, Huazhong University of Science and Technology, 1277# Jiefang Avenue, Wuhan, 430022 Hubei People’s Republic of China

**Keywords:** CircESRP1, Endometrial cancer, miR-874-3p, CPEB4, EMT, Proliferation

## Abstract

**Background:**

Metastasis is critical for endometrial cancer (EC) progression and prognosis. Accumulating evidence suggests that circular RNAs (circRNAs) can operate as independent functional entities. However, the functional regulatory mechanisms of circRNAs in EC remain unclear.

**Methods:**

The levels of circESRP1, miR-874-3p, and CPEB4 mRNA in EC tissues and cells were determined by qRT-PCR. Sanger sequencing, PCR with divergent primers, an actinomycin D assay, and RNase R treatment were applied to verify the circular properties. Fluorescence in situ hybridization (FISH) and nuclear-cytoplasmic fractionation were used to determine the localization of circESRP1. CCK-8, EdU incorporation, colony formation, Transwell, and wound healing assays were applied to assess the effects of circESRP1 on cell proliferation, migration, and invasion. The mutual regulatory mechanism of ceRNAs was investigated using dual-luciferase reporter, RNA pulldown, RNA immunoprecipitation (RIP), and Western blot assays. The biological effects were further validated in vivo in nude mouse xenograft models.

**Results:**

circESRP1 was highly expressed in EC tissues and cells and was mainly localized in the cytoplasm. Silencing circESRP1 inhibited the proliferation, migration, and invasion of EC cells in vitro and in vivo; however, overexpression of circESRP1 had the opposite effects. Mechanistically, circESRP1 sponged miR-874-3p to upregulate CPEB4 expression and ultimately contribute to EC cell proliferation and metastasis. Furthermore, circESRP1 regulated tumour growth in xenograft models.

**Conclusions:**

CircESRP1 can interact with miR-874-3p to regulate EMT in endometrial cancer via the miR-874-3p/CPEB4 axis. CircESRP1 may serve as a promising therapeutic target for endometrial cancer.

**Supplementary Information:**

The online version contains supplementary material available at 10.1186/s12967-022-03334-6.

## Background

Endometrial cancer (EC) is the sixth most common gynaecologic malignancy and threatens women’s health and life, increasing the disease risk and medical burden on society. In 2020, there were 417,000 new cases of endometrial cancer and 97,000 deaths from EC worldwide; its morbidity was 8.2% and mortality was 1.4% in Eastern Asia [1]. The overall 5-year survival rate of patients with endometrial cancer is approximately 80% [2, 3]. However, the clinical prognosis of advanced and specific subtypes of EC, such as high-grade EC and EC with papillary serous or clear cell histology, is extremely poor. Unexpected recurrence and poor outcomes with early-stage or well-differentiated endometrioid tumors do occur [4]. Surgery, endocrine therapy, chemotherapy, radiotherapy, and immunotherapy are the principal treatments for endometrial cancer [5]. Hence, it is pivotal to identify new molecular targets and molecular mechanisms to facilitate improved diagnosis and treatment of EC.

Epithelial-to-mesenchymal transition (EMT) is a process that is activated during cancer progression, causing cancer cells to acquire mesenchymal or stem cell properties that allow them to detach from the primary tumour site and invade surrounding tissues or vascular tissues to form distant metastases [6, 7]. Loss of E-cadherin expression and acquisition of Vimentin expression are markers of EMT occurrence [8]. Research has shown that EMT is critically associated with tumour metastasis in the development of tumorigenesis. LncRNA MAFG-AS1 mediates NFKB1-induced upregulation of IGF1 through miR-339-5p to promote EMT in ovarian cancer [9]. In renal clear cell carcinoma, E2F1 modulates SREBP1 to induce cellular lipid accumulation and elevated lipase, thereby promoting tumour cell proliferation and metastasis [10]. DLX6-AS1 contained in hepatocarcinoma exosomes modulates CXCL17 by binding competitively to miR-15a-5p and triggers M2 macrophage polarization, promoting EMT in hepatocarcinoma [11]. The EMT process is primarily correlated with the Wnt signalling pathway, Notch signalling pathway, and Hedgehog signalling pathway, which are involved in tumour cell metastasis [12]. SRA is engaged in EMT in EC cells by enhancing the expression of EIF4E-BP1 and activating the Wnt/β-catenin signalling pathway [13]. The cytoplasmic polyadenylation element-binding protein (CPEB) family consists of sequence-specific RNA-binding proteins. CPEB1, CPEB2, CPEB3, and CPEB4 are four members of the CPEB family [14]. The CPEB family is differentially expressed and activated in different tumours. CPEB4 has been identified as playing an important role in the proliferation of tumour cells and tumour development progression [15–18]. Increasing evidence has shown that CPEB4 is involved in tumour invasion and metastasis. For example, CPEB4 can regulate tumour cell invasion and migration by regulating Vimentin expression in breast cancer; ZEB1-mediated EMT may be involved in CPEB4-promoted cell proliferation, invasion, and metastasis in gastric cancer [19, 20]. However, the pathophysiological roles of CPEB4 in EC remain uninvestigated. Therefore, further studies are of paramount importance to determine the mechanism underlying CPEB4-driven EMT in EC.

Circular RNAs (circRNAs) are a type of closed-loop noncoding RNA without a 5′ cap or 3′ poly (A) tail [21, 22]. They were initially regarded as the products of splicing errors, but they are now considered independent functional entities [23, 24]. CircRNAs play important roles in many aspects of tumorigenesis, such as proliferation, metastasis, apoptosis, and autophagy [25]. CircRNAs can also function as sponges for miRNAs that influence the mRNA translation process [26]. The roles of circRNAs as microRNA (miRNA) sponges in EC are being gradually discovered. Circ_0002577, circ_PUM1, circTNFRSF21, circRNA WHSC1, circ_0002577, and circ_0067835 have been reported to influence the biological function of EC by sponging miRNAs [27–32]. However, the roles of most circRNAs in EC remain uninvestigated.

In this study, bioinformatics analysis was performed to investigate the expression of circRNAs in EC tissue. The expression level of hsa_circ_0084927 (circESRP1), which is derived from the ESRP1 gene locus, was markedly upregulated in EC tissues. Up- or downregulation of circESRP1 correspondingly promoted or inhibited tumour cell proliferation and migration. CircESRP1 was demonstrated to sponge miR-874-3p, which degrades CPEB4 mRNA. In conclusion, circESRP1 regulates EMT and proliferation in EC by acting as a ceRNA to bind to miRNA-874-3p. CircESRP1 may serve as a novel biomarker for the diagnosis and prognosis of EC and may be a potential therapeutic target for EC.

## Methods

### Human tissues sample and databases

Human normal endometrial tissues (n = 10) and EC tissues (n = 19) were obtained from Union Hospital, Tongji Medical College, Huazhong University of Science and Technology (Wuhan, China). The tissue specimens were all from patients undergoing surgery and were removed and stored in liquid nitrogen until proteins or RNAs were extracted. Patients read and signed an informed consent form before the surgery. Institutional Review Board of the Tongji Medical College, Huazhong University of Science and Technology approved this research.

### Quantitative real-time polymerase chain reaction (qRT-PCR)

Total RNA from cells or fresh tissues was extracted using TRizol (#9108, RNAiso Plus, Takara, Japan) according to the instructions. The cDNA was prepared by using PrimeScriptRT Reagent Kit (#RR037A, Takara, Japan). qRT-PCR was performed by the Biosystem StepOne Plus PCR System (ABI) with Real-time PCR kits (Takara, Japan). The RNA expression levels were calculated using GAPDH and U6 expression as internal references by the 2−ΔΔCT method. The sense and antisense primers of qRT-PCR were shown as follows: circESRP1 5′-GGAACGGAGAAGCTCTGGTTAG-3′ and 5′-GTAAACCTCGTGCCCTGACTAC-3′; linear ESRP1 5′-ACCAAGCCCTCCGACAGTTTA-3′ and 5′-ATCAGGTGAACCAGGGCAACA-3′; miR-874-3p 5′-CTGCCCTGGCCCGAGG-3′ and RT primer 5′-GTCGTATCCAGTGCAGGGTCCGAGGTATTCGCACTGGATACGACTCGGTC-3′; miR-4694-5p 5′-CAAATGGACAGGATAACACCTATG-3′ and RT primer 5′-GTCGTATCCAGTGCAGGGTCCGAGGTATTCGCACTGGATACGACCAAATA-3′; miR-4732-3p 5′-TGTCCTGTTCTGCCCCCAG-3′ and RT primer 5′-GTCGTATCCAGTGCAGGGTCCGAGGTATTCGCACTGGATACGACGAGGGG-3′; miR antisense primers 5′-AGTGCAGGGTCCGAGGTATT-3′; CPEB4 5′-TGTGTGCTACGCTGGGATTGA-3′ and 5′-TGGCTTAACTTCCACCCGTTT-3′; U6 5′-CTCGCTTCGGCAGCACA-3′ and 5′-AACGCTTCACGAATTTGCGT-3′; GAPDH 5′-GGCAGAGATGATGACCCTTTT-3′ and 5′-AGATCCCTCCAAAATCAAGTGG-3′.

### Cell culture

EC cell lines included RL95-2 and Ishikawa, which were purchased from the American Type Culture Collection (ATCC; Manassas, VA). Ishikawa cells and RL952 cells were cultured in DMEM/F12 medium (#11330032, Gibco) supplemented with 10% fetal bovine serum (#10099141, Gibco) and 1% streptomycin and penicillin (#PYG0016, Bosterbio, USA) in the 5% CO_2_, 37 °C incubator.

### Transfection

Lentivirus vector containing short hairpin RNA (shRNA) and overexpression lentivirus were purchased from GenChem (Shanghai, China). Three shRNA sequences of circ0084927 were: sh-circ0084924-1: 5′-CTTAAAATTGCTGGTGCAGCA-3′; sh-circ0084927-2: 5′-AAAATTGCTGGTGCAGCAAGA-3′; sh-circ0084927-3: 5′-TGCTGGTGCAGCAAGATGGAA-3′; sh-negative control (sh-NC): 5′-TTCTCCGAACGTGTCACGT-3′. Lentiviral transfection of cells was performed when cell integration reached 20–30%. MiR-874-3p mimic/mimic control/inhibitor/inhibitor control were designed and purchased from RiboBio (Guangzhou, China). Transfection of miR-874-3p mimic, miR-874-3p mimic control, miR-874-3p inhibitor, and miR-874-3p inhibitor control was performed when EC cells were melted in 6-well plates with 60–70% conformation utilizing co-transfection reagents (Guangzhou, China). CPEB4 three small interfering RNAs (shRNA) and negative control shRNA were designed and synthesized by GenChem (Shanghai, China). The target sequences of CPEB4 were described as follows:

- sh-CPEB4-1, 5′-TAGCCGATCCATTATCATC-3′;
- sh-CPEB4-2, 5′-ATACTGTTCAACACATAAC-3′;
- sh-CPEB4-3, 5′-ATATTATACCAAAGCGCAG-3′.

### Cell counting kit-8 assay

Cell Counting Kit-8 assay (#34302, CCK8, Bimake, USA) was used to detect cell proliferation. Cells (4 × 10^3^/well) were cultivated in 96-well plates, and the absorbance at 450 nm was detected at four-time points of 24 h, 48 h, 72 h, and 96 h according to the instructions. The absorbance was performed by using an automatic microplate reader (BioTek, VT, USA).

### EdU assay

Cells (1 × 10^4^ cells/well) were grown in 96-well plates and incubated at 37 °C, 5% CO_2_ thermostat for 24 h before EdU (5-ethynyl-2′-deoxyuridine) assay. EdU assay was measured using an EdU kit (#C0071S, Beyotime, shanghai) in accordance with the instructions. The EdU solution was prepared as a 1:1000 EdU medium. Add 50 µl/well of EdU medium to the pre-prepared 96-well plate and the cells were incubated at 37 °C for 2 h and then fixed using 4% paraformaldehyde for 15 min. Cells were incubated with 30 µl/well of click reaction solution for 30 min at room temperature and sheltered from light, and then Hochst33342 stained for 10 min. Cell proliferation was imaged using a 20× microscope, and 5 isolated fields were photographed for counting statistics.

### Colony formation assay

Cells (1 × 10^3^ cells/well) were seeded in 6-well plates and cultured at 37 °C with 5% CO_2_ for 3 weeks. Cells were fixed with 4% paraformaldehyde for 15 min and then stained with 0.1% crystal violet for 25 min. Clone number and size were used to assess the ability of cell clone formation, and five separate fields of view were used for enumeration.

### Migration and invasion assays

Migration assays were performed in 24-well plates. 600 µl of medium containing 20% fetal bovine serum was added to the lower part of the transwell chamber (#3422, Corning, USA) and 200 µl of serum-free medium containing 10 × 10^4^ cells was added to the upper part of the transwell chamber. Following 24 h of incubation at 37 °C, cells penetrated from the top of the chamber membrane (8 μm pore size) to the bottom. Cells penetrating the chamber membrane were fixed with 4% paraformaldehyde for 15 min and stained with 0.1% crystal violet for 30 min, followed by imaging under a 10× microscope, and four separate fields of view were taken for counting. In contrast to the cell migration assay, the cell invasion assay requires the addition of Matrigel (#356234, Corning, USA) to the upper part of the cell chamber before seeding the cells, and the same steps as the cell migration assay.

### Wound healing assay

Cells were implanted in six-well plates and cultured for 24 h to reach 100% fusion. Cell wounds were made by a 10 µl pipette tip, and cells were rinsed clean by PBS. The cells were continued to be cultured in a serum-free medium, and the width of the wound was photographed at 0 h and 24 h under a 40× magnification microscope.

### RNase R treatment and actinomycin D

Total RNA (2 μg) was incubated with or without 3 U/μg of RNase at 37 °C for 45 min. For the actinomycin D treatment assay, total RNA was obtained using 2 mg/μl of actinomycin D after treating the cells for 0 h, 4 h, 8 h, and 12 h. After actinomycin D and RNAase treatment, qRT-PCR was operated to evaluate variations in the expression of circESRP1 and ESRP1 mRNA.

### Nuclear-cytoplasmic fraction

Cytoplasmic and nuclear RNA isolation was performed by utilizing the Cytoplasmic and nuclear RNA purification kit (#21000, Norgenbiotek, Canada). Cells were washed twice with PBS and 200 µl of pre-cooled Lysis buffer J was added and incubated on ice for 5 min. The cell lysate was centrifuged at 12,000 rpm for 10 min at 4 °C to separate the supernatant of the cytoplasmic fraction from the nuclear pellet. The cytoplasmic supernatant and the nuclear pellet were washed sequentially by vortexing using Buffer K and 100% ethanol and subsequently transferred to a centrifuge column for centrifugation. The RNA solution was obtained by washing the centrifuge column 3 times with wash solution A and then lysing the RNA with 50 µl of Elution Buffer E.

### Fluorescence in situ hybridization

Cy3-labeled circ0084927, human U6, and human18S probes were designed and synthesized by RiboBioCo. Ltd. (Guangzhou, China). The FISH assay was performed using the RiBoTM Fluorescent In Situ Hybridization Kit (#C10910, RiboBio Co. Ltd, Guangzhou, China) according to the instructions. Cells (1 × 10^4^/well) were replanted in 24-well plates and incubated in the incubator for 24 h before the FISH assay. Cells were treated with PBS 2 times and then fixed using 4% paraformaldehyde at room temperature for 10 min, followed by permeabilization with 0.5% Triton-X100 at 4 °C for 5 min. After 30 min of pre-hybridization at 37 °C, cells were hybridized with the fluorescent probe overnight at 37 °C sheltered from light. Thereafter, cells were rinsed 3 times/5 min using 4×/2×/1× SCC solution at 42 °C under light-protected conditions and then stained with DAPI. The cells were eventually observed utilizing confocal microscopyat 400× magnification.

### RNA pulldown

The biotinylated-circ0084927 probe was synthesized by Sangon Biotech (Shanghai, China). The cells were harvested and lysed and subsequently centrifuged, and the lysate was collected. The cell lysates were incubated with a circ0084927 probe and an oligo probe for 2 h at room temperature. Then, the cell lysates incorporating the probes were incubated with the streptavidin magnetic beads (#HY-K0208, MedChemExpress, USA) for 1 h at room temperature. The RNA bound to the magnetic beads was then purified and withdrawn using RNeasy Mini Kit (Qiagen, German). Lastly, qRT-PCR experiments were performed.

### RNA immunoprecipitation

RNA binding protein immunoprecipitation (RIP) assay was performed with the EZ-Magna RIP kit (#17-704, Millipore, Burlington, MA, USA) based on the guidelines. The purpose of RIP was to extract and identify the RNA bound to AGO2 protein. Cells (1 × 10^7^) were added to the lysis solution, lysed on ice for 5 min, and then centrifuged, and the supernatant was extracted. After incubation with AGO2 antibody and IgG antibody for 30 min, the magnetic beads were combined with 100 μl of cell lysate supernatant, respectively, rotated 360° and incubated overnight at 4 °C. After washing the magnetic bead protein RNA complex, RNA was extracted and qRT-PCR was performed.

### Dual-luciferase reporter assay

Fragments of wild-type and mutant circ0084927 and CPEB4 were constructed and inserted downstream of the reporter plasmid pRL-SV40 with firefly fluorescence (GenChem, Shanghai, China). Cells were seeded in 6-well plates, and when cell fusion reached 50%, the plasmids and miR-874-3p mimic or controls were transfected using lipo3000. After 48 h of incubation, firefly luciferase and renilla luciferase activities were detected using the Dual-Luciferase Reporter System kit (#E1910, Promega, USA).

### Western Blot (WB) analysis

Total cellular proteins were extracted with RIPA lysate (#P0013B, Beyotime, Shanghai, China) and the proteins were measured quantitatively using the BCA protein assay kit (#G2026, Servicebio, Wuhan, China). Total cellular protein (30 µg) was pipetted into 10% or 12.5% SDS-PAGE gel for gel electrophoresis and subsequently transferred to polyvinylidene difluoride membranes. The membranes were incubated with primary antibodies overnight at 4 °C after 2 h of blocking at room temperature using 5% skim milk. Membranes were washed three times with TBST buffer and incubated with goat anti-rabbit or goat anti-generic secondary antibodies for 1 h at room temperature. Finally, imaging was performed using The ChemiDoc MP (Bio-Rad, USA). The primary antibodies were as follows: CPEB4 (1:1000, #25342-1-AP, Proteintech Group, INC., USA), CRCP (1:1000, #14348-1-A, ProteintechGroup, INC., USA), Vimentin (1:1000, #10366-1-AP, Proteintech Group, INC., USA), E-cadherin (1:1000, #20874-1-AP, Proteintech Group, INC., USA), GAPDH (1:20,000, #AC002, ABclonal, Wuhan, China); secondary antibodies are as follows: HRP Goat Anti-Rabbit IgG (1:8000, #AS014, ABclonal, Wuhan, China), HRP Goat Anti-Mouse IgG (1:8000, #AS003, ABclonal, Wuhan, China), China).

### Immunohistochemistry

Paraffin sections of mouse subcutaneous graft tumor tissue were dewaxed and dehydrated, rehydrated, and repaired with citric acid. After peroxidase blocking, the tissue sections were incubated with primary antibody overnight at 4 °C. The primary antibodies were as follows: CPEB4 (1:2000, #25342-1-AP, Proteintech Group, INC., USA), Vimentin (1:2000, #10366-1-AP, Proteintech Group, INC., USA), E-cadherin (1:3000, #20874-1-AP, Proteintech Group, INC., USA). The sections were washed with PBS and incubated with secondary antibody for 30 min at 37 °C. The sections were finally blocked and observed under the microscope. According to the staining score, the staining intensity was categorized as negative (score = 0), weak (score = 1), moderate (score = 2), and strong (score = 3); the number of positively stained cells 0–5%, 5–25%, 26–50%, 51–75%, and 76–100% were scored as 0, 1, 2, 3, 4, respectively. The final staining score was the product of the staining intensity and the number of positively stained cells products.

### Hematoxylin–eosin staining (HE staining)

Xylene I and II were used to dewax the sections for 10 min each. 100% (I and II), 90%, 80% and 70% alcohol were used to dehydrate the sections for 5 min each and rinsed under running water for 5 min × 3. Hematoxylin was utilized to stain the sections for 5 min and rinsed under running water. Acetic acid fractionation for 1 min, rinse slides with running water. Eosin staining for 1 min, rinsing the slides under running water. 70%, 80%, 90%, 100% alcohol for 10 s each, xylene for 1 min, dehydrating the slides. Slides were dried naturally and sealed with drops of neutral gum.

### In vivo tumor xenografts

All animals in experiments were approved and supported by the Animal Ethics Committee of Tongji Medical College, Huazhong University of Science and Technology. 6-week-old female BALB/c nude mice were purchased from SPF Biotechnology Co., Ltd. (Beijing, China), and Ishikawa cells (4 × 10^6^ per mouse) were injected subcutaneously into the back of mice. Mice were housed for 28 days in a specific pathogen-free class of animal room. The sizes of the mice subcutaneous tumors were measured weekly. Tumor size was measured and mice were sacrificed, and tumors were removed and weighed. The tumor volume was calculated by the formula: a × b^2^, where a indicated the longest diameter of the tumor and b was the length of the diameter perpendicular to a.

### Database

The differential circRNA data in EC were extracted from the analysis of EC tissue samples by the team of Yongchao Dou, which consisted of 95 cases of EC and 49 normal tissue samples [4]. Subsequently, the differential circRNAs were performed using R software, where the parameters were set fold change ≥ 2 and p_adj_ ≤ 0.001.

### Statistical analysis

The data in this study, which were from 3 independent replicate experiments, were analyzed by GraphPadPrism software (version 7.0), and the results are expressed as mean ± SD. The significance of differences between groups was assessed by paired two-tailed Student’s t-test or χ^2^ test. Correlation analysis between circESRP1 and miR-874-3p was evaluated by Spearman’s test. A p-value   0.05 was admitted as statistically significant.

## Results

### Characterization of circESRP1 in EC

**Fig. 1:**
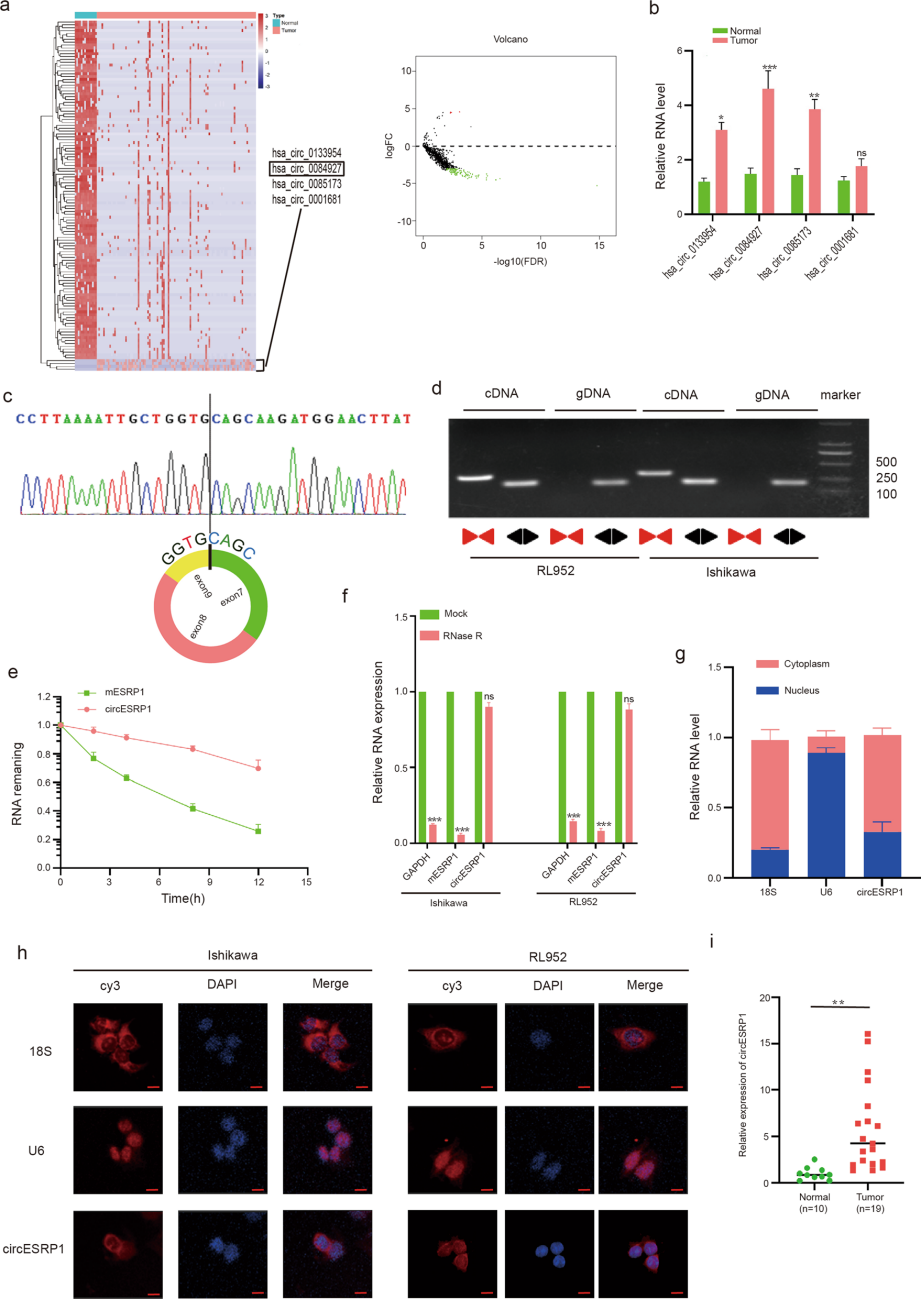
Characterization of circESRP1 in EC. **a** CircRNAs with significant upregulation were selected based on the criteria of fold change > 2 and P value < 0.01 by using a heatmap and volcano plot. In the volcano plot, red dots indicate circRNAs with increased expression in EC, and green dots indicate circRNAs with decreased expression in EC. **b** The expression of the differentially highly expressed circRNAs was validated in EC and normal tissues by RT-qPCR. **c** The figure illustrates that circESRP1 is backspliced into a loop by exons 7–9 of ESRP1, and Sanger sequencing confirms the back-spliced junction sites. **d** The presence of circESRP1 was verified by qRT-PCR in Ishikawa cells and RL952 cells. Different primers amplified circESRP1 in cDNA rather than genomic DNA (gDNA). **e** The relative RNA levels of Ishikawa cells upon exposure to actinomycin D were analysed by qRT-PCR. **f** The relative RNA levels of Ishikawa cells and RL952 cells treated with or without RNase R were analysed by qRT-PCR. **g** Cytoplasmic and nuclear distribution of circESRP1 in Ishikawa cells was analysed by qRT-PCR. 18S and U6 served as positive controls for the cytoplasm and nucleus, respectively. **h** Identification of circESRP1 location by FISH in Ishikawa cells and RL952 cells. 18S and U6 were used as positive controls in the cytoplasm and nucleus, respectively. **i** qRT-PCR analysis of circESRP1 in tumour tissues and normal endometrial tissues. Scale bar: 10 μm. n = 3, *P < 0.05, **P < 0.01, ***P < 0.001

Bioinformatics analysis was used to filter out 692 differentially expressed circRNAs in EC tissues compared to normal tissues. A fold change of > 2.0 and a P value of < 0.05 were used as assessment criteria to identify significantly differentially expressed circRNAs. hsa_circ_0133954, hsa_circ_0084927, hsa_circ_0085173, and hsa_circ_0001681 were upregulated, as shown in the heatmap and volcano plot (Fig. 1a). The expression patterns of these four circRNAs were verified in EC tissues (10 cases) and adjacent tissues (10 cases) by qRT-PCR (Fig. 1b). CircESRP1, which has never been reported, was expressed at significantly higher levels in EC tissues than in normal tissues (Fig. 1b). According to circBase (http://www.circbase.org/), circESRP1 was derived from ESRP1, whose backsplice junction sequence was amplified with divergent primers and validated by Sanger sequencing (Fig. 1c). The head-to-tail splicing of endogenous circESRP1 was detected by qRT-PCR using polymeric primers and divergent primers. circESRP1 could be amplified by divergent primers in cDNA but not in genomic DNA (gDNA), as expected (Fig. 1d). The transcripts of circESRP1 were more stable after actinomycin D treatment than ESRP1 mRNA (Fig. 1e). Moreover, the resistance to the digestive action of RNase R exonuclease confirms that circESRP1 has a circular RNA structure (Fig. 1f). In addition, the results of qRT-PCR analysis of cytosolic/nuclear RNA differences and FISH showed that circESRP1 is mainly localized in the cytoplasm (Fig. 1g, h). Figure 1i indicated that the expression of circESRP1 was significantly higher in endometrial cancer tissues than in normal tissues. Moreover, there was no correlation between the expression of circESRP1 and the stage of endometrial cancer (Additional file 1: Fig. S1a).

Altogether, these findings determined that circESRP1 was a bona fide circRNA predominantly distributed in the cytoplasm and significantly upregulated in EC cells.

### Knockdown of circESRP1 suppressed EC cell proliferation, migration, and invasion

**Fig. 2:**
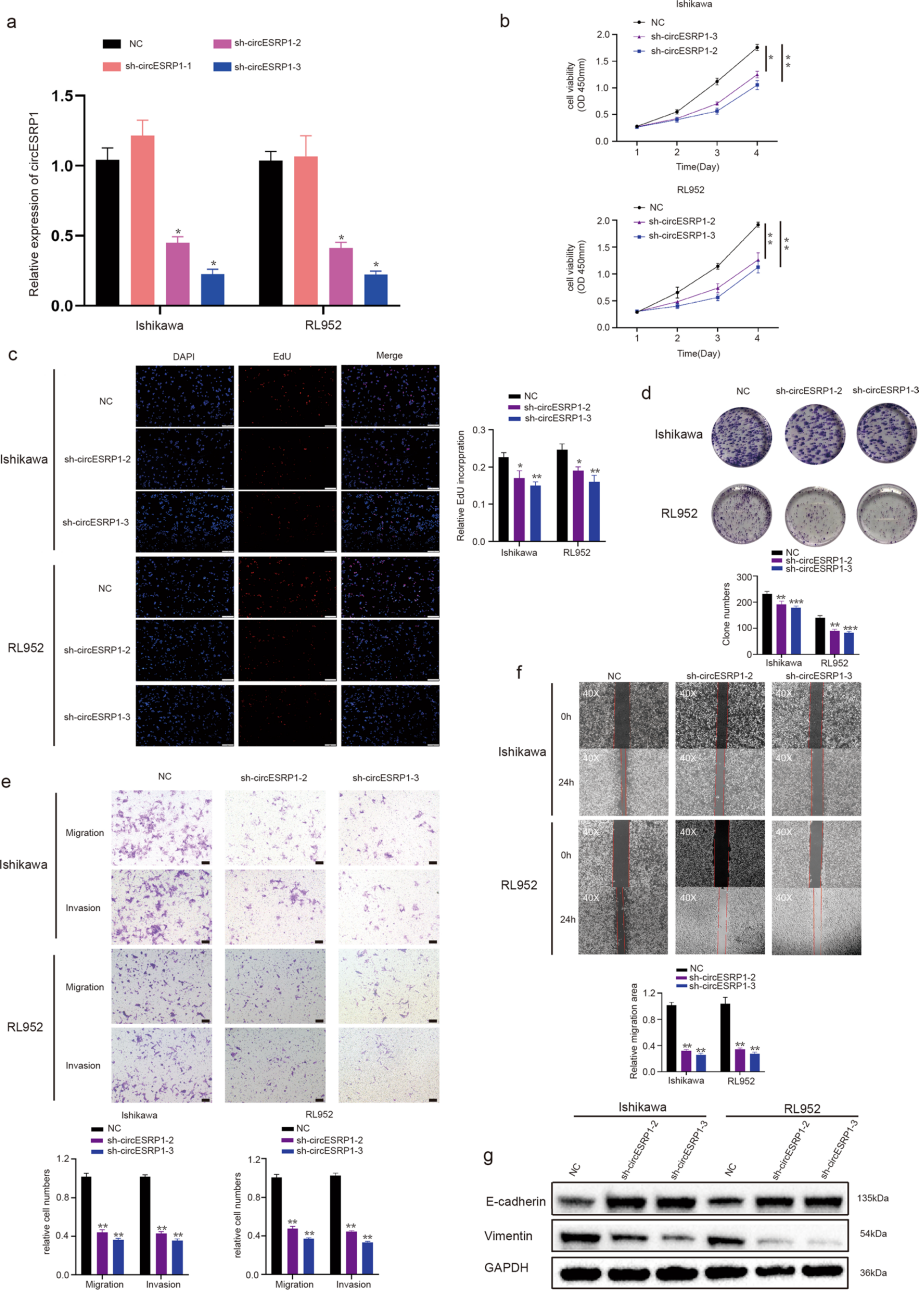
Knockdown of circESRP1 suppressed EMT and EC cell proliferation, migration, and invasion. **a** qRT-PCR analysis of circESRP1 expression in Ishikawa cells and RL952 cells after transfection of the expression shRNA. **b**–**d** CCK-8, EdU incorporation, and colony formation assay were carried out to assess the proliferation of Ishikawa cells and RL952 cells. **e**, **f** Transwell and wound healing assays (magnification, ×40) were performed to assess the migration and invasion of Ishikawa cells and RL952 cells. **g** EMT-related proteins (E-cadherin and Vimentin) were detected by Western blot analysis in Ishikawa cells and RL952 cells transfected with sh-circESRP1. Scale bar: 100 μm. n = 3, *P < 0.05, **P < 0.01, ***P < 0.001

To further investigate the role of circESRP1 in EC development, short hairpin RNAs (shRNAs) were used to manipulate the levels of circESRP1 in Ishikawa cells and RL952 cells, aiming to study its effects on cancer cell proliferation and migration. We demonstrated that cells transfected with sh-circESRP1-2 and sh-circESRP1-3 had much lower circESRP1 levels than cells transfected with sh-negative controls (NC) (Fig. 2a). CCK-8, EdU incorporation, and colony formation assay were used to investigate cell proliferation. The CCK-8 product, EdU admixture rate, and the number of colonies formed revealed that the proliferation ability of Ishikawa cells and RL95-2 cells was reduced when circESRP1 was knocked down, indicating that knockdown of circESRP1 inhibits EC cell proliferation (Fig. 2b–d). The Transwell and wound healing assays showed that knockdown of circESRP1 significantly reduced the number of EC cells (Fig. 2e, f). EMT is an important phenotype in tumour metastasis. Therefore, we investigated whether circESRP1 affected EMT in EC cells. WB analysis showed that knockdown of circESRP1 in EC cells increased the expression of the epithelial marker E-cadherin and decreased the expression of the mesenchymal marker Vimentin (Fig. 2g), suggesting that knockdown of circESRP1 inhibited the EMT process. Taken together, we demonstrated that downregulation of circESRP1 suppressed the EMT process and inhibited the proliferation, migration, and invasion of EC cells.

### CircESRP1 promoted the proliferation, migration, and invasion abilities of EC cells in vitro

**Fig. 3:**
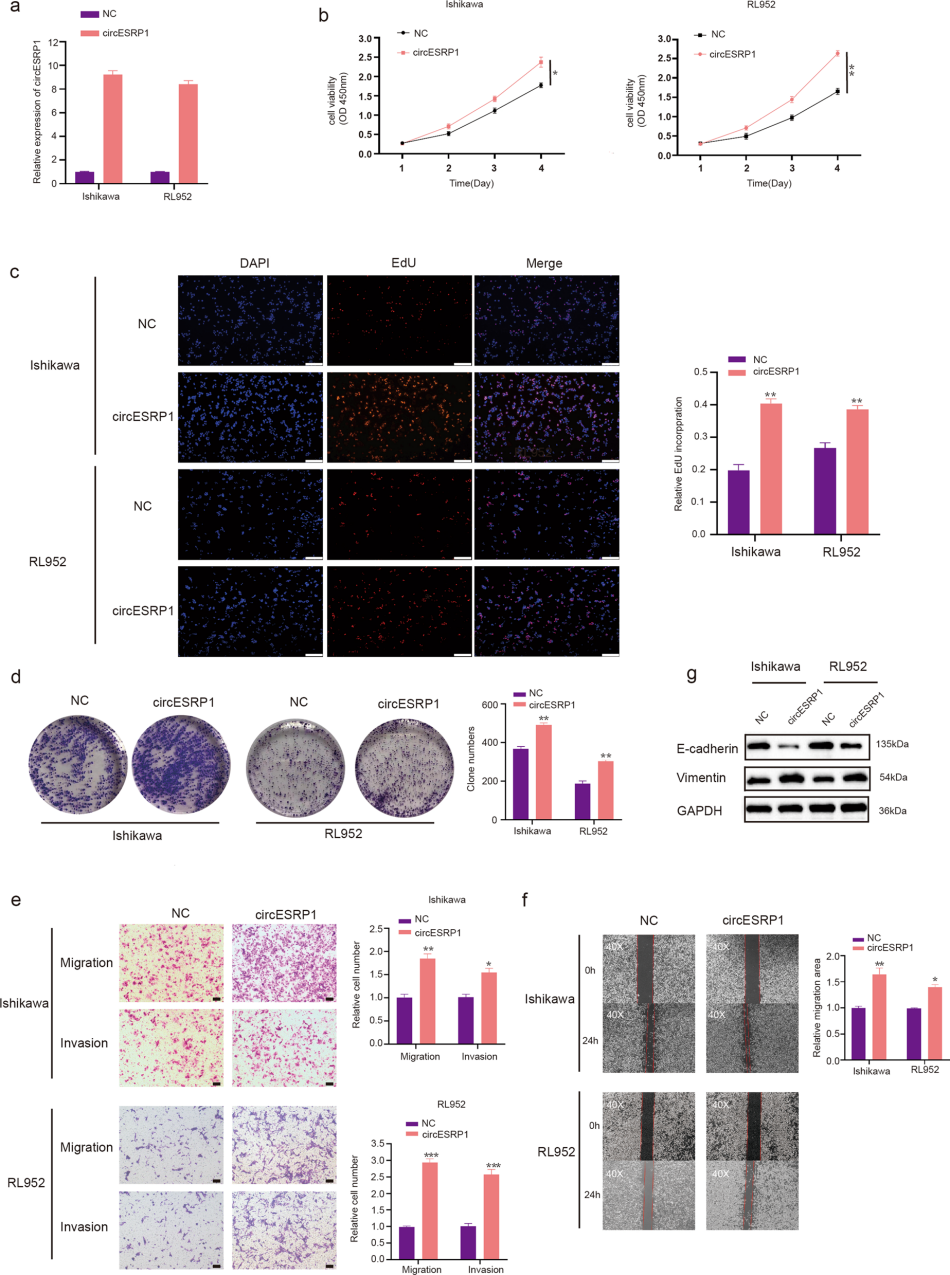
CircESRP1 promoted the proliferation, migration, and invasion abilities of EC cells in vitro. **a** qRT-PCR analysis of circESRP1 expression in Ishikawa cells and RL952 cells after transfection of the expression vector. **b**–**d** CCK-8, EdU incorporation, and colony formation assay were carried out to assess the proliferation of Ishikawa cells and RL952 cells. **e**, **f** Transwell and wound healing assays (magnification, ×40) were performed to assess the migration and invasion of Ishikawa cells and RL952 cells. **g** EMT-related proteins (E-cadherin and Vimentin) were detected by Western blot analysis in Ishikawa cells and RL952 cells transfected with the circESRP1 vector. Scale bar: 100 μm. n = 3, *P < 0.05, **P < 0.01, ***P < 0.001

To reconfirm the biological function of circESRP1 in EC cells, a circESRP1 overexpression vector was constructed, and significant increases in expression following transfection of the circESRP1 overexpression vector were verified using qRT-PCR in Ishikawa cells and RL952 cells (Fig. 3a). CCK-8, EdU incorporation, and colony formation assay showed that the proliferation abilities of Ishikawa cells and RL95-2 cells were enhanced when circESRP1 was overexpressed (Fig. 3b–d). The results of the Transwell assays revealed that the migration and invasion abilities of Ishikawa cells and RL952 cells were significantly enhanced when the expression of circESRP1 was increased (Fig. 3e). The wound healing assay showed the same conclusion as the Transwell assays (Fig. 3f). Furthermore, the WB results revealed that EC cells transfected with the circESRP1 overexpression vector had a significantly higher level of Vimentin but a lower level of E-cadherin than control NC-transfected cells (Fig. 3g).

In summary, we concluded that circESRP1 promoted EC cell proliferation and facilitated EC cell migration and invasive ability through EMT.

### CircESRP1 acts as a sponge for miR-874-3p

**Fig. 4:**
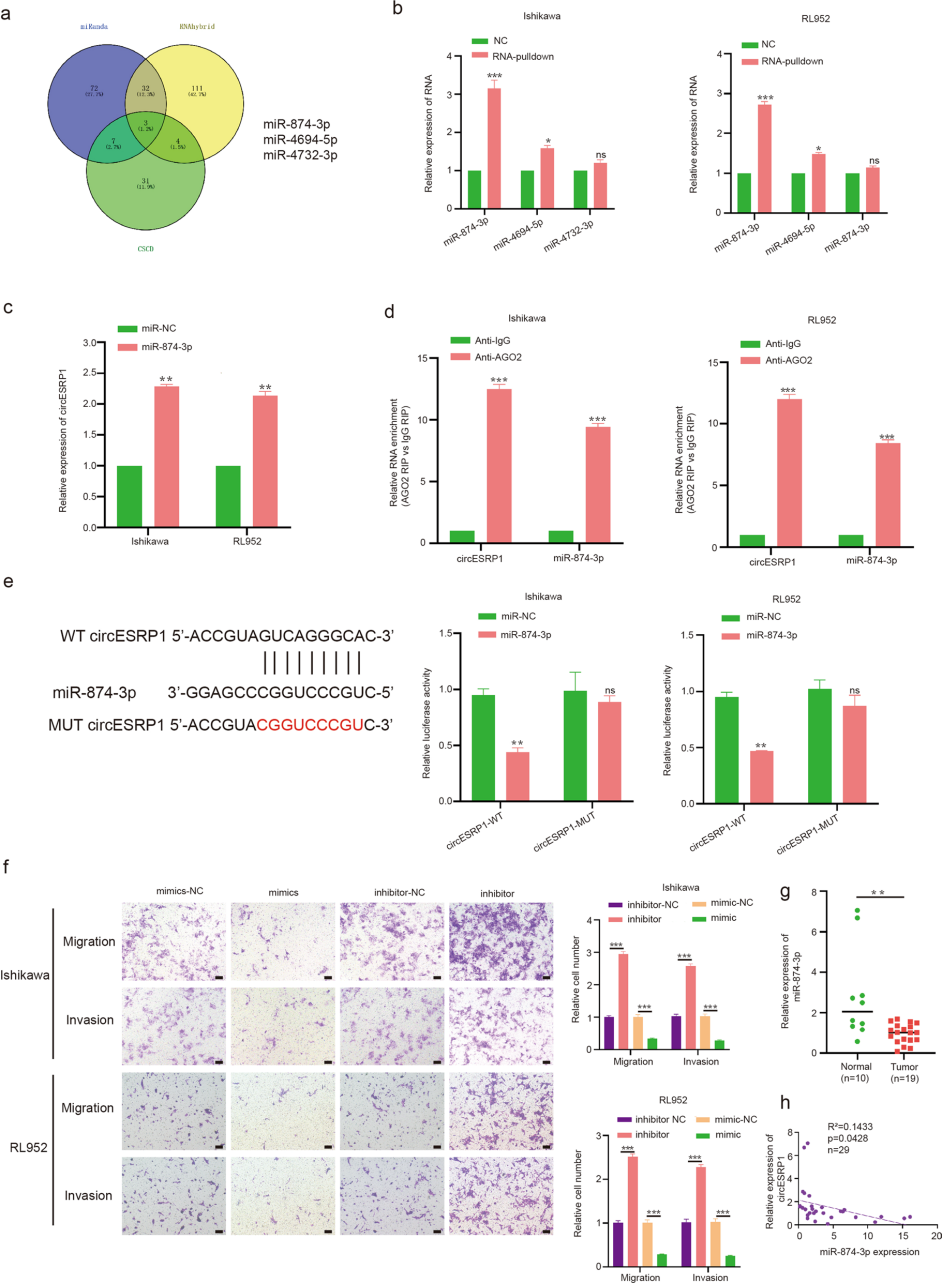
CircESRP1 acts as a sponge for miR-874-3p. **a** Venn diagram suggested that 3 miRNAs might be targets of circESRP1 sponge action. **b**, **c** RNA pulldown was performed in Ishikawa cells and RL952 cells, followed by qRT-PCR to detect the enrichment of circESRP1 and related miRNAs. **d** Anti-AGO2 RIP was performed in Ishikawa cells and RL952 cells, followed by qRT-PCR to detect the capacity for AGO2 enrichment on circESRP1 and miR-874-3p compared to IgG. **e** The luciferase reporter assay functionally validated the interaction between circESRP1 and miR-874-3p in in Ishikawa cells and RL952 cells. **f** Transwell assays demonstrated the effects of the miR-874-3p mimic and inhibitor on Ishikawa cells and RL952 cell migration and invasion. **g** The relative RNA levels of miR-874-3p in EC tissues and normal tissues were determined by qRT-PCR. **h** The negative interaction between miR-874-3p and circESRP1. Scale bar: 100 μm, n = 3, *P < 0.05, **P < 0.01, ***P < 0.001

Next, we investigated the molecular mechanisms underlying circESRP1 function. Previous studies suggested that circRNAs consisting of exons and mainly distributed in the cytoplasm may function as miRNA sponges [33]. Bioinformatics assays of circESRP1 were performed using three online databases (miRanda: http://www.microrna.org/microrna/home.do, RNAhybrid: http://bibiserv.techfak.uni-bielefeld.de/rnahybrid/, CSCD: http://gb.whu.edu.cn/CSCD/), and three miRNA (hsa-miR-874-3p, hsa-miR-4694-5p, hsa-miR-4732-3p) alternatives interacting with circESRP1 were selected as candidate miRNAs for subsequent experiments (Fig. 4a). To further verify which miRNA interacts with circESRP1, an RNA pulldown assay was performed in Ishikawa cells and RL952 cells. MiR-874-3p was pulled down more than the other two miRNAs in Ishikawa cells and RL952 cells by immunoprecipitation with a biotin-labelled probe, which targeted the circESRP1 back-spliced sequence (Fig. 4b). In contrast, specific enrichment of circESRP1 was detected by qRT-PCR in the miR-874-3p probe group compared with the control probe, which was consistent with previous results (Fig. 4c). To reconfirm that the biological behaviour of EC cells was regulated by circESRP1 through a ceRNA mechanism, a RIP assay was performed. It is known that the critical component of the RNA-induced silencing complex (RISC) is Argonaute2 (AGO2), which is essential for miRNAs to regulate the functions of target genes. Therefore, an anti-AGO2 RIP assay was conducted in Ishikawa cells and RL952 cells. An anti-AGO2 antibody was used as the experimental treatment to pull down the RNA transcripts binding to AGO2, and IgG was used as the negative control. The results showed that circESRP1 and miR-874-3p were pulled down more abundantly in the AGO2 group than in the IgG group (Fig. 4d). To further verify the targeting relationship between circESRP1 and miR-874-3p, a dual-luciferase reporter gene assay was carried out. The full-length circESRP1-WT and mutant versions without miR-874-3p binding sites were inserted into the luciferase reporter vector psiCHECK2. The outcomes revealed that luciferase activity was reduced more in the circESRP1-WT group than in the mutant group by the miR-874-3p mimic. The foregoing results suggested that there may be a direct interaction between circESRP1 and miR-874-3p (Fig. 4e). EMT-related functional experiments using the miRNA mimic and inhibitor were performed in Ishikawa cells and RL952 cells. The results revealed that the miR-874-3p mimic could inhibit the invasion and migration ability of EC cells, whereas the miR-874-3p inhibitor had the opposite effect (Fig. 4f). The RNA level of miR-874-3p in EC tissues and normal tissues was verified using qRT-PCR. The results showed that the expression in normal tissues was higher than that in cancer tissues, and a negative correlation was observed between the RNA levels of circESRP1 and miR-874-3p (Fig. 4g, h). Therefore, we concluded that circESRP1 may function as a miR-874-3p sponge in EC.

### MiR-874-3p targets CPEB4 in EC cells

**Fig. 5:**
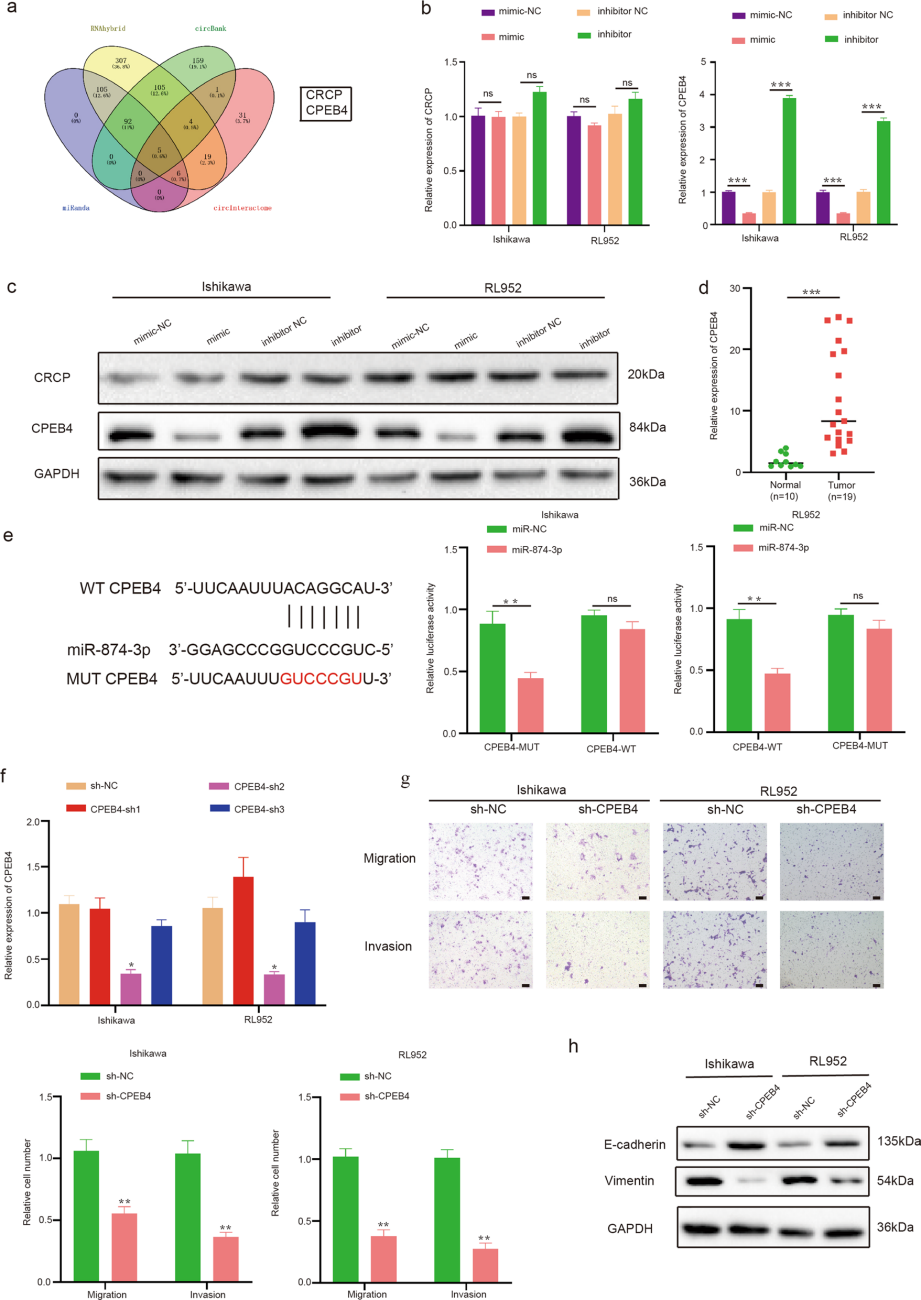
MiR-874-3p targets CPEB4 in EC cells. **a** Venn diagram suggested that 2 genes might be targets of miR-874-3p. **b**, **c** The relative RNA and protein levels of CRCP and CPEB4 were determined by qRT-PCR and Western blot analyses, respectively, after transfection with miR-874-3p mimic and inhibitor in Ishikawa cells and RL952 cells. **d** The relative RNA levels of CPEB4 in EC tissues and normal tissues were determined by qRT-PCR. **e** The luciferase reporter assay functionally validated the interaction between miR-874-3p and CPEB4 in Ishikawa cells and RL952 cells. **f** The CPEB4 expression in Ishikawa cells and RL952 cells after transfection of shRNA was determined by qRT-PCR analysis. **g** The impacts of CPEB4 knockdown on the migration and invasion of Ishikawa cells and RL952 cells were examined by using Transwell assays. **h** The expression of EMT-related proteins was detected by Western blot analysis. Scale bar: 100 μm, n = 3, *P < 0.05, **P < 0.01, ***P < 0.001

To further explore the role of circESRP1 in EC progression and the target genes of miR-874-3p, four online databases (miRanda, RNAhybrid, circBank, and circInteractome) were utilized for prediction. In accordance with the bioinformatics analysis results, two genes (CRCP and CPEB4) were screened for further validation (Fig. 5a). qRT-PCR and WB analyses were conducted after transfection of Ishikawa cells and RL952 cells with the miR-874-3p mimic, miR-874-3p inhibitor, or the corresponding negative control. The results indicated that the mRNA and protein levels of CPEB4 but not CRCP were significantly reduced by the miR-874-3p mimic. Conversely, the miR-874-3p inhibitor showed the opposite result (Fig. 5b, c). Finally, the level of CPEB4 mRNA in EC tissues and normal tissues was determined by qRT-PCR. The results demonstrated that the mRNA level of CPEB4 was higher in EC tissues than in normal tissues (Fig. 5d). The luciferase reporter assay showed that the activity of the luciferase reporter containing the wild-type CPEB4 sequence (WT) was significantly decreased by the miR-874-3p mimic; however, this effect was eliminated upon mutation of the miR-874-3p binding site (Fig. 5e). To evaluate the function of CPEB4 in EC cells, Ishikawa cells and RL952 cells were transfected with short hairpin RNAs (shRNAs) targeting CPEB4 (including shCPEB4-1, sh-CPEB4-2, and sh-CPEB4-3) or the corresponding negative control (Fig. 5f). The Transwell assay indicated that silencing CPEB4 expression suppressed both the migration and invasion of Ishikawa cells and RL952 cells (Fig. 5g). In addition, the WB analysis results showed that EMT in Ishikawa cells and RL952 cells was inhibited by knockdown of CPEB4 (Fig. 5h).

### The circESRP1/miR-874-3p/CPEB4 axis promoted EC progression by activating EMT

**Fig. 6:**
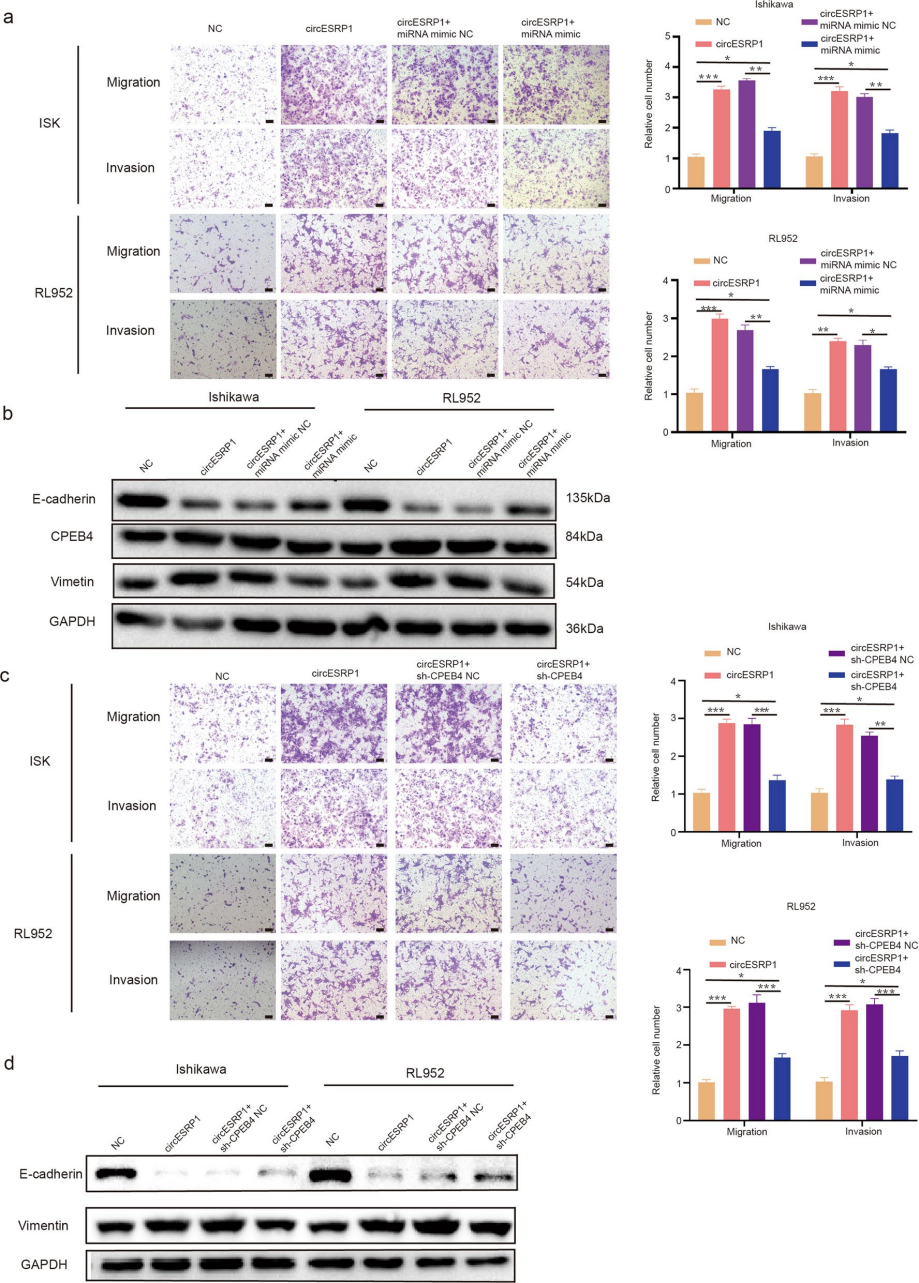
The circESRP1/miR-874-3p/CPEB4 axis promoted EC progression by activating EMT. **a** The Transwell assay indicated that the migration and invasion abilities of Ishikawa cells and RL952 cells transfected with the circESRP1 vector were counteracted when the cells were cotransfected with the miR-874-3p mimic.** b** Western blot analysis showed that the levels of EMT-related proteins and CPEB4 transfected with the circESRP1 vector were rescued when Ishikawa cells and RL952 cells were cotransfected with the miR-874-3p mimic. **c** The results of Transwell assays indicated that sh-CPEB4 inhibited the promotive effect of circESRP1 on Ishikawa cells and RL952 cell metastasis. **d** Western blot analysis showed that the level of EMT-related proteins transfected with circESRP1 vector was rescued when Ishikawa cells and RL952 cells were cotransfected with sh-CPEB4. Scale bar: 100 μm, n = 3, *P < 0.05, **P < 0.01, ***P < 0.001

Prior experimental results revealed that circESRP1 can regulate CPEB4 expression and thus affect EMT in EC cells by sponging miR-874-3p. To further confirm the ceRNA pathway between the three components, rescue experiments were performed using the miR-874-3p mimic and sh-CPEB4. The Transwell assay results revealed that miR-874-3p mimic transfection and CPEB4 silencing reversed the effects on migration and invasion induced by overexpression of circESRP1 in Ishikawa cells and RL952 cells (Fig. 6a, c). Moreover, WB analysis showed that the protein levels of Vimentin and CPEB4 were enhanced by upregulation of circESRP1, which diminished the protein level of E-cadherin. The effects induced by overexpression of circESRP1 could be reversed by the miR-874-3p mimic and sh-CPEB4 (Fig. 6b, d). To further validate that circESRP1 regulates endometrial cancer EMT via CPEB4, simultaneous silencing of circESRP1 and CPEB4 was performed in Ishikawa cells and RL952 cells. The results of Transwell assays suggested a stronger inhibitory effect was observed with simultaneous knockdown of circESPR1 and CPEB4 than with knockdown of circESRP1 or CPEB4 alone (Additional file 1: Fig. S1b). The results of WB were consistent with the trend of Transwell assay results (Additional file 1: Fig. S1c). The protein level of Vimentin was significantly decreased in the sh-circESRP1-3 + sh-CPEB4 group, while the protein level of E-cadherin was significantly elevated.

In summary, these results suggested that circESRP1 may act as a ceRNA for miR-874-3p to regulate CPEB4 expression, leading to the migration, invasion, and development of EC.

### CircESRP1 enhances tumour growth and metastasis in vivo

**Fig. 7:**
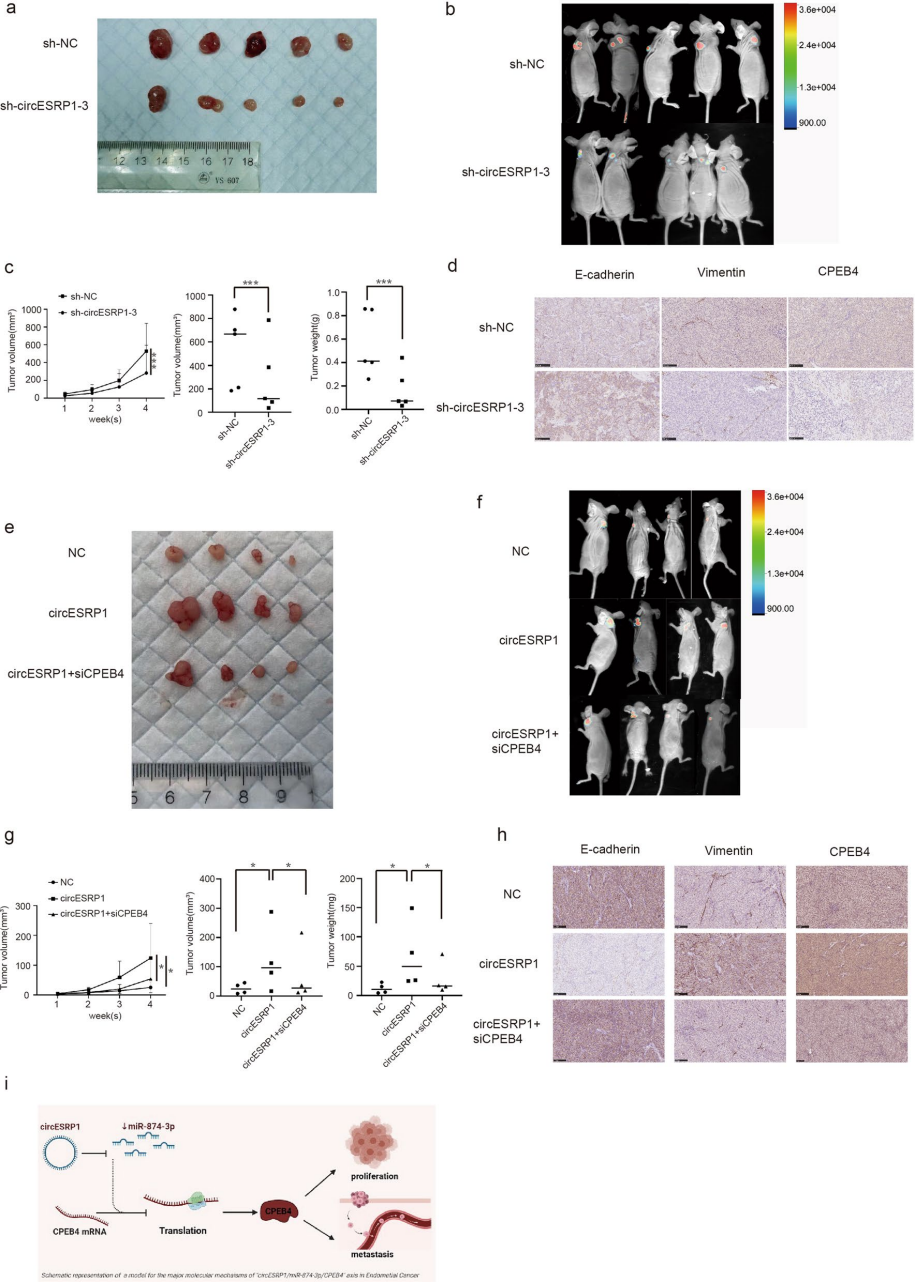
CircESRP1 enhances tumour growth and metastasis in vivo. **a**, **e** Images of subcutaneous injection of BALB/c nude mice. **b**,** f** Images of xenograft tumours of each group. **c**, **g** Tumour volume and weight measurement in BALB/c nude mice. **d**, **h** The relative protein levels of E-cadherin, Vimentin, and CPEB4 were determined in subcutaneous xenograft tumours by IHC. Scale bar, 100 μm. n = 3, *P < 0.05, **P < 0.01, ***P < 0.001

To explore the influence of circESRP1 on the growth and metastasis of EC cells in vivo, two groups of animal experiments were performed. One group of Ishikawa cells stably transfected with sh-circESRP1 or sh-NC vector and the other group of Ishikawa cells stably transfected with circESRP1 overexpression vector, circESRP1 overexpression control (NC) and circESRP1 overexpression vector with sh-CPEB4 were constructed. The stably transfected cells were injected subcutaneously into female BALB/c nude mice, and the relevant indices of the tumours were measured once a week and taken out after 4 weeks. HE staining was used to show the cellular morphology of subcutaneous graft tumours (Additional file 1: Fig. S1d, e). The results indicated that the tumour sizes and weights in the sh-circESRP1 group were significantly smaller than those in the sh-NC group (Fig. 7a–c). Moreover, these subcutaneous tumours were subjected to immunohistochemical staining, which showed that compared with those in the sh-NC group, the protein levels of CPEB4 and Vimentin were significantly decreased in the sh-circESRP1 group, while the protein level of E-cadherin was significantly elevated (Fig. 7d). The tumours sizes and weights in the circESRP1 overexpression vector group were significantly larger than those in the NC group, and these differences were partially reversed by cotransfection with sh-CPEB4 (Fig. 7e–g). Similarly, these subcutaneous tumours were subjected to immunohistochemical staining, and the results showed that the protein levels of CPEB4 and Vimentin were significantly upregulated in the circESRP1 overexpression vector group and that cotransfection with sh-CPEB4 partially reversed the effects, while the protein level of E-cadherin showed the opposite trend (Fig. 7h). In conclusion, these results demonstrated that circESRP1 may regulate EC cell proliferation, migration, and invasion in vivo.

## Discussion

Although the 5-year survival rate of EC is 80%, its annual morbidity is rising and threatens women’s health [31]. Accumulating evidence indicates that circRNAs are involved in tumorigenesis and tumour development and may potentially serve as tumour markers in the future [34]. Based on bioinformatics analysis, we selected circESRP1, a 287 bp exonic circRNA (EcircRNA) containing three exons from its parental gene ESRP1, to elucidate its biological and potential mechanisms. CircESRP1 is a novel circRNA that exhibited the most significant differences in expression among all circRNAs. Our study investigated the function and mechanism of circESRP1 in EC for the first time. Utilizing qRT-PCR, we found that the RNA level of circESRP1 was higher in EC tissues than in adjacent tissues. However, the lower RNA level of miR-874-3p in EC, which has been reported to be associated with tumour progression, was verified by qRT-PCR. These results suggest that the differential expression of circESRP1 and miR-874-3p may be significant for the diagnosis of EC and may serve as biomarkers of EC. Current research indicates that dysregulation of circRNA expression may contribute to progressive, uncontrolled tumour cell growth and metastasis [35, 36], not simply a nonfunctional product of pre-mRNA splicing. Functionally, silencing circESRP1 reduced the proliferation and invasion abilities of EC cells, and conversely, overexpression of circESRP1 had enhancing effects in vitro and in vivo. Therefore, our data suggested that circESRP1 may play a key role in pathogenesis and assessing the progression of EC, providing a basis for being a prospective therapeutic target in the future.

Regarding circRNAs containing miRNA response elements, a vast number of studies have reported that miRNA adsorption is the most pivotal pathway by which circRNAs perform their biological functions, which can further modulate downstream target genes, thus influencing malignant tumour progression, recurrence, and chemoresistance [22]. For example, circSPARC upregulates JAK2 expression by sponging miR-485-3p, leading to accumulation of phosphorylated p-STAT, which in turn promotes CRC cell proliferation and metastasis [37]; in addition, circPDE3B can induce EMT to regulate ESCC cell proliferation, migration, and invasion through the miR-4766-5p/LAMA1 axis [38]. Through the experiment, circESRP1 was identified as a miRNA sponge to promote EC cell progression. Bioinformatics analysis predicted that circESRP1 interacts with miR-874-3p, and the anti-AGO2 RIP, RNA pulldown, and dual-luciferase reporter assays confirmed our speculation. Our study revealed that miR-874-3p was expressed at low levels in EC, and rescue experiments reconfirmed that circESRP1 exerted its oncogenic effect through miR-874-3p. In papillary thyroid carcinoma, miR-874-3p suppressed tumour cell migration and invasion by downregulating the expression of FAM84A and served the same function in colon cancer, [39, 40] consistent with our findings that the miR-874-3p mimic inhibited EC cell metastasis and that the miR-874-3p inhibitor enhanced EC cell function. This study shows for the first time that circESRP1 and miR-874-3p interact and that circESRP1 promotes EC cell progression via miR-874-3p. These results also indicate that miR-874-3p plays an important role in the progression of EC and may be a potential therapeutic target.

In addition, bioinformatics analysis and validation experiments indicated that miR-874-3p binds to CPEB4, a member of the CPEB family. CPEB4 is intimately associated with the metastasis of a variety of tumours. For example, CPEB4 can promote breast cancer metastasis through upregulation of Vimentin [19] and facilitate the migration and invasion of lung cancer cells through activation of the AKT pathway [41]. Thus, CPEB4 could enhance the metastasis of tumour cells. In our study, we detected upregulation of CPEB4 in EC tissues. Knockdown of CPEB4 inhibited migration, invasion, and corresponding changes in EMT-related proteins in EC cells, whereas the rescue experiments suggested that cotransfection of the circESRP1 vector partially attenuated these inhibitory effects. CircESRP1 overexpression increased CPEB4 expression, while the miR-874-3p mimic partially abolished these promotive effects. These results suggest that circESRP1 acts as a sponge for miR-874-3p to regulate CPEB4 and promote EMT, metastasis, and proliferation in EC.

## Conclusion

Our results indicate that circESRP1 is upregulated in EC tissues, promoting EC cell tumorigenesis and metastasis by sponging miR-874-3p to promote CPEB4 expression. The circESRP1/miR-874-3p/CPEB4 axis may serve as a therapeutic target for EC.

## Supplementary Information

**Additional file 1: Figure S1.** **a** Expression of circESRP1 in different stages of endometrial cancer. **b** The Transwell assay indicated that the migration and invasion abilities of Ishikawa cells and RL952 cells cotransfected with the sh-circESRP1-3 and sh-CPEB4 were suppressed more significantly than the cells were transfected with the sh-circESRP1-3 or sh-CPEB4 alone. **c** Western blot analysis showed that the levels of EMT-related proteins were consistent with the trend of the Transwell assay. **d**, **e** Subcutaneous xenograft tumours by HE. Scale bar, 100 μm. n = 3, *P < 0.05, **P < 0.01, ***P < 0.001.

## Data Availability

Not applicable.

## Abbreviations

EC: Endometrial cancer
FISH: Fluorescence in situ hybridization
CCK-8: Cell Counting Kit-8 Assay
RIP: RNA immunoprecipitation
EMT: Epithelial-to-mesenchymal transition
EdU: 5-Ethynyl-2′-deoxyuridine
CPEB: Cytoplasmic polyadenylation element-binding protein
