# Supplementary figures and images for: CircESRP1 enhances metastasis and epithelial–mesenchymal transition in endometrial cancer via the miR-874-3p/CPEB4 axis
Source: J Transl Med. 2022 Mar 22;20:139. doi: 10.1186/s12967-022-03334-6 (PMC8939068; doi:10.1186/s12967-022-03334-6)

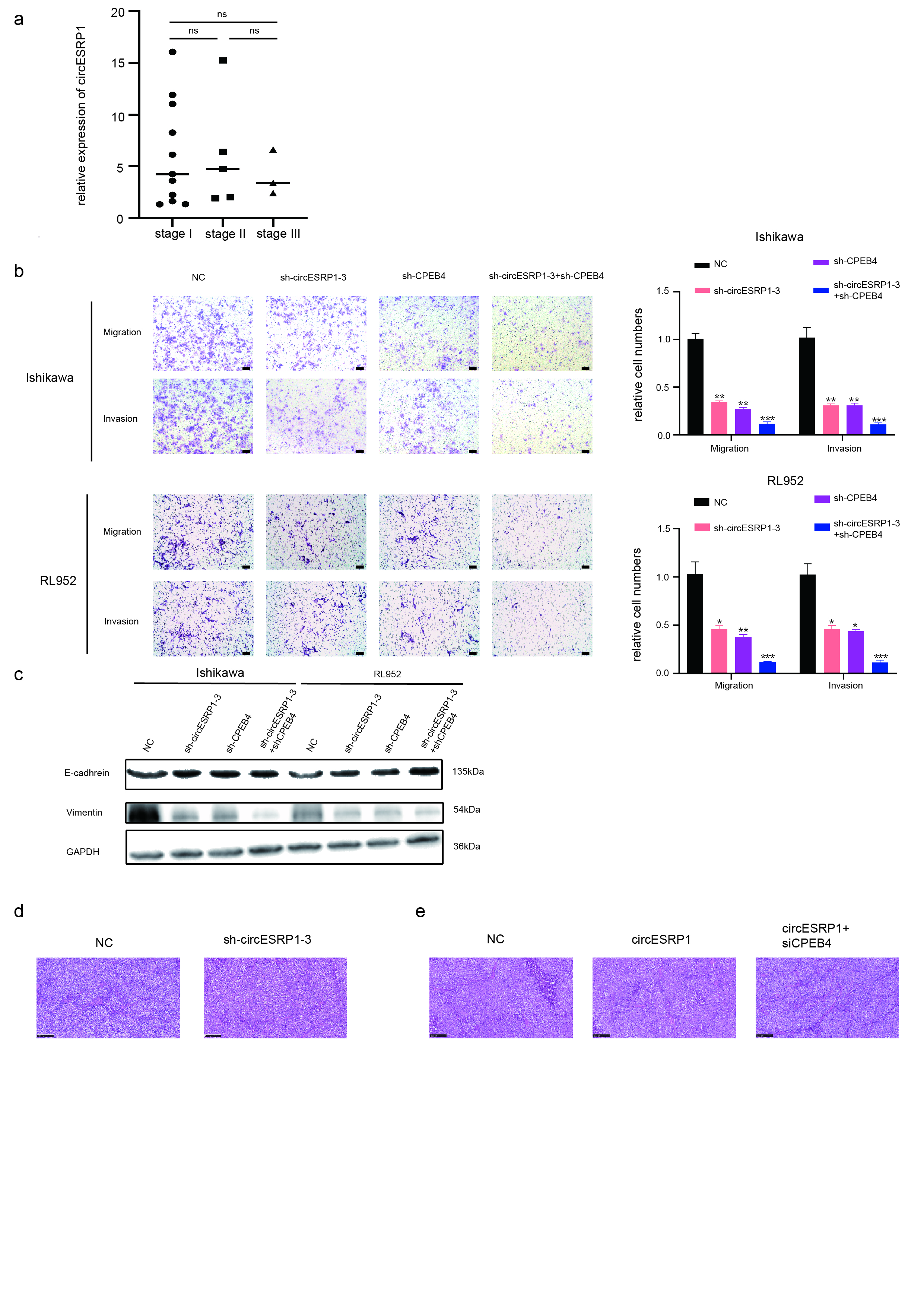

Supplement: Supplementary file 1 — Additional file 1: Figure S1. a Expression of circESRP1 in different stages of endometrial cancer. b The Transwell assay indicated that the migration and invasion abilities of Ishikawa cells and RL952 cells cotransfected with the sh-circESRP1-3 and sh-CPEB4 were suppressed more significantly than the cells were transfected with the sh-circESRP1-3 or sh-CPEB4 alone. c Western blot analysis showed that the levels of EMT-related proteins were consistent with the trend of the Transwell assay. d, e Subcutaneous xenograft tumours by HE. Scale bar, 100 μm. n = 3, *P < 0.05, **P < 0.01, ***P < 0.001. [file 12967_2022_3334_MOESM1_ESM.jpg]
